# The disquietude of clozapine continuation during the COVID‐19 pandemic

**DOI:** 10.1002/hsr2.506

**Published:** 2022-02-07

**Authors:** Sheikh Shoib, Vidya Bharati‐Sinha, Sana Javed, Ahmet Gürcan, Gamze Gürcan, Soumitra Das, Miyuru Chandradasa, Fahimeh Saeed

**Affiliations:** ^1^ Department of Psychiatry Jawahar Lal Nehru Memorial Hospital Srinagar Kashmir India; ^2^ Department of Psychiatry Shri Krishna Medical College and Hospital Muzaffarpur India; ^3^ Nishtar Medical University Multan Pakistan; ^4^ Department of Psychiatry Başkent University Medical Faculty Ankara Turkey; ^5^ Department of Psychiatry Akdağmadeni State Hospital Yozgat Turkey; ^6^ North Western Mental Health Melbourne Health Melbourne Australia; ^7^ Department of Psychiatry University of Kelaniya Ragama Sri Lanka; ^8^ Department of Psychiatry Psychosis Research Centre, University of Social Welfare and Rehabilitation Sciences Tehran Iran

The coronavirus disease (COVID‐19) pandemic has posed numerous challenges to the world. Patients who suffer from a mental health condition are more likely to become infected with COVID‐19 because they may have difficulties in following precautionary measures, needing regular contact with a health facility for psychological monitoring and a reduction in health care support during the pandemic.^1^ The risk of infection is also increased because of comorbidities and/or psychotropic medication side effects.^1^ In a recent systematic review of sixteen studies conducted in seven nations, higher COVID‐19 mortality was seen in people with mental disorders.^2^ Therefore, individuals with psychiatric disorders should be considered as high‐risk for severe COVID‐19 complications and encourage to take substantive preventive strategies.

Clozapine is an important medicine in the treatment of resistant schizophrenia.^3^ Although effective, clozapine has a wide range of significant side effects that some of them are irritating such as sedation, dizziness, gastrointestinal symptoms, sialorrhea, benign fever etc. Some of them are life threatening such as seizures, heart adverse effects, pneumonia, hepatic failure, pancreatitis, respiratory failure, agranulocytosis, and sudden death.^4^ Many side effects overlap with COVID‐19 symptoms such as fever, flu‐like symptoms, and myalgia that is seen with neutropenic sepsis (Table 1).^5^, ^6^ Further, in a study conducted in the United Kingdom among 6309 persons with schizophrenia, it was found that individuals on clozapine had an increased risk of COVID‐19 infection compared with those who were on other antipsychotics.^7^ It is consistent with previous studies showing clozapine is associated with higher rates of infection and pneumonia than those on other antipsychotics.^8^, ^9^ It is thought that the higher incidence of pneumonia is likely due to sialorrhea. As a result, it raises the risk of pneumonia with COVID‐19 infection.^10^

**TABLE 1:** The clozapine side effects in overlap with coronavirus disease 2019 (COVID‐19)

Organ	Symptoms
Heart	Cardiomyopathy, myocarditis, pericarditis, tachycardia
Lung	Pneumonia
Central nervous system	Seizure, headache, dizziness
Blood and coagulation system	Agranulocytosis, thromboembolism
Gastrointestinal	Nausea, vomiting, bloating, stomach pain
Flu‐like symptoms	Fever, myalgia, fatigue, headache

Persons on clozapine are at risk for cardiovascular events and death due to the presence of comorbid medical disorders.^11^ Diabetes and cardiovascular disease are common comorbidities that enhance the risk. Myocarditis, pericarditis, and cardiomyopathy could be consequence of both COVID‐19 and clozapine. These adverse cardiac events appear with highly variable symptoms from a flu‐like initiation to chest pain, shortness of breath, fever, tachypnea, and sudden death. Serial MB isoenzyme of creatine phosphokinase (CPK‐MB), C‐reactive protein (CRP), troponin test, and ECG is advised in order to determine any sign which refers to a cardiac event.^12^, ^13^ Clozapine‐induced cardiac complications may be missed and delayed presenting to services due to the similarity with the symptomatology of the COVID‐19. A delay could lead to serious consequences such as clozapine‐induced myocarditis has an estimated mortality of more than 20%.^14^

Fever in patients who take clozapine is related to varied causes from a benign fever to serious condition such as neutropenic sepsis.^3^ To reduce the risks of concealing COVID‐19 symptoms, it is advisable to avoid paracetamol treatment for clozapine fever. To avoid neutropenic sepsis, a white blood cells (WBC) count with absolute neutrophil count monitoring is required. It will help to distinguish between neutropenic sepsis and COVID‐19 infection if symptoms appear. Vitamin D supplementation is necessary to reduce the risk of pneumonia, and smoking cessation should be promoted.^15^ Diabetes is a common side effect, and it raises the risk of COVID‐19 infection and related complications. Blood glucose monitoring is particularly crucial.^3^

A cumulative 10% risk of seizures after 3.8 years of clozapine treatment has been recognised.^16^ COVID‐19 infection has been linked to a variety of neurological symptoms, including headache, dizziness, myalgia and anosmia, encephalopathy, encephalitis, necrotizing hemorrhagic encephalopathy, stroke, epileptic seizures, rhabdomyolysis, and Guillain‐Barre syndrome.^17^ COVID‐19 infection increases the risk of thromboembolism and raises the risk of deep venous thrombosis and pulmonary embolism.^18^ Venous thromboembolism is a rare clozapine adverse effect, although smoking, weight gain, and cardiovascular disease all enhance the risk.^19^ When a person on clozapine contracts COVID‐19, it is likely that neurological and vascular complications become more likely than the general population.

Many medicines are used off‐label for the treatment of COVID‐19. Chloroquine, hydroxychloroquine, azithromycin, lopinavir/ritonavir, ribavirin, remdesivir, and tocilizumab are the most used medications. Each of these substances distinctly interacts with psychotropic drugs. With clozapine, atazanavir, lopinavir/ritonavir, and QT prolongation can occur, necessitating strict monitoring.^20^ Azithromycin causes neutropenia, which is also a common clozapine side effect.^21^ Clozapine has been reported to increase ribavirin blood levels due to pharmacokinetic interactions, which increases the risk of bone marrow suppression and necessitates comprehensive blood count monitoring.^15^ Ribavirin produces serious adverse effects, including depression, aggressiveness, and suicide ideation. Due to a reduction in metabolism in response to inflammatory reactions, COVID‐19 infection enhances the probability of clozapine toxicity.^22^ Clozapine produces constipation, nausea, vomiting, bloating, and stomach pain due to its anticholinergic impact on the gastrointestinal system.^3^, ^23^ The medications used to treat COVID‐19 also have gastrointestinal adverse effects that include nausea, vomiting, and diarrhoea that may add to the suffering in the affected patient.

Access to the vaccination may be difficult for persons with mental disorders, and antipsychotics, which is known to cause immune dysfunction may impact the vaccination efficacy.^24^ There is also a report of elevated clozapine levels following administration of an mRNA vaccine.^25^ Long‐term clozapine use can result in secondary antibody deficiency and low class‐switched memory B cells.^26^ Substantive information on COVID‐19 vaccination and the immune response while on clozapine therapy is not available at present.

Mental health care delivery, access to clozapine tablets, and availability of monitoring investigations are arduous during the pandemic. Clozapine dispensation can be stopped if absolute neutrophils are not done and reported during an infective wave or a lockdown. Patients may not have access to current lab work due to a lack of transportation, inability to leave the house due to the danger of infection, or lab closures in resource‐limited locations.^27^, ^28^ If testing is difficult to come by, the neutrophil count can be done every 3 months, and drug dispensation can be done every 90 days if the patient has been on medication for more than a year, and there has been no history of neutropenia below 2000/μL.^29^ Telehealth, caregiver training, online resource education, and the activation of warm lines are all essential in this situation.^27^, ^28^

Continuing clozapine is related to better occupational activity, independent living, and low rate of hospitalization and relapse, as well as good outcome in suicidal individuals.^30^, ^31^ According to several side effects that are confusing in time of pandemic and increasing risk of COVID‐19, the prescription and discontinuation of clozapine is a dilemma. Before beginning treatment of clozapine, the risk and benefit should be carefully examined, considering the drug's potential side effects and the need for constant monitoring.^3^ The patients must be closely monitored. Tachycardia, hypotension, fever, and sedation are frequent side effects of clozapine medication that do not always require medication termination. In this situation, frequent vital sign monitoring is required, which may be difficult owing to contact confines. In certain situations, clozapine could be sustained even combined with immunosuppression treatment with a precise assessment and close monitoring.^32^ The early diagnosis of rare but life‐threatening side effects and clozapine suspension is essential.

## FUNDING

None.

## CONFLICT OF INTEREST

The authors declare that there is no conflict of interest.

## AUTHOR CONTRIBUTION

Conceptualization and writing original draft: Sheikh Shoib.

Writing ‐ review and editing: All the authors worked equally.

## TRANSPARENCY STATEMENT

Authors confirm that manuscript is an honest, accurate, and transparent account of the study being reported; that no important aspects of the study have been omitted; and that any discrepancies from the study as planned (and, if relevant, registered) have been explained.
